# Comparison of Whole Exome Sequencing Commercial Kits Performance Across Diverse Tissue Sources

**DOI:** 10.3390/ijms27146261

**Published:** 2026-07-14

**Authors:** Edgard Verdura, Aina Montalbán-Casafont, Xavier Solé, Esther Titos, Eva González-Roca, Cristina Sánchez-Cárdenas, Vanessa López, Núria Palau, Paula Sánchez, Alfredo Mendoza-Cantero, Laura Rodriguez-Elena, Dolores Jiménez, Abraham José Paredes-Fuentes, Laura Gort, Laia Rodríguez-Revenga, Irene Madrigal, Yolanda López-Púa, Judit García-Villoria, Celia Badenas, José Luis Villanueva-Cañas, Joan Anton Puig-Butillé

**Affiliations:** 1 Molecular Biology CORE, Hospital Clínic de Barcelona, 08036 Barcelona, Spain; 2 Division of Molecular Genetics, Biochemistry and Molecular Genetics Department, Hospital Clínic de Barcelona, 08036 Barcelona, Spain; 3 Faculty of Medicine and Health Sciences, University of Barcelona (UB), 08036 Barcelona, Spain; 4 Fundació de Recerca Clínic Barcelona, Institut d’Investigacions Biomèdiques August Pi i Sunyer (FCRB-IDIBAPS), 08036 Barcelona, Spainjugarcia@clinic.cat (J.G.-V.); 5 Immunology Department, Hospital Clínic de Barcelona, 08036 Barcelona, Spain; 6 Center for Biomedical Network Research on Rare Diseases (CIBERER), Instituto de Salud Carlos III, 08036 Barcelona, Spain; 7 Division of Inborn Errors of Metabolism—IBC, Department of Biochemistry and Molecular Genetics, 08036 Barcelona, Spain; 8 Quality Unit, Biomedical Diagnostic Center, Hospital Clinic de Barcelona, 08036 Barcelona, Spain

**Keywords:** exome, coverage, uniformity, Twist, Agilent, Illumina, NGS

## Abstract

Whole exome sequencing (WES) is a standard, relevant diagnostic strategy in medical genetics. However, commercial WES library preparation kits vary significantly in design and covered regions. Very few studies have assessed DNA tissue of origin’s impact on WES performance, which relates to preanalytical factors that may compromise DNA quality. We evaluated three WES commercial solutions using eight DNA samples extracted from peripheral blood, amniotic fluid, fetal tissue, and fibroblasts. A second set of 24 previously sequenced samples was resequenced using the highest-performing kit to validate results. A comprehensive bioinformatics pipeline was applied to evaluate key metrics such as coverage uniformity, duplication rates, or estimated library size, among others. Twist Exome 2.0 capture kit demonstrated the best overall performance, achieving more uniform coverage, lower duplication rates (8% vs. 16–17%), and a larger estimated library size (243 M vs. 102–129 M). Additionally, this kit achieved 93% of regions covered at the standard threshold of 38x (compared to ~80% in the other two solutions). Notably, Twist Exome 2.0 capture consistently delivered superior results for DNA extracted from non-peripheral blood tissues. This study suggests that Twist Exome 2.0 provides robust performance and is particularly well-suited for samples where DNA quality may be compromised due to preanalytical limitations.

## 1. Introduction

Whole exome sequencing (WES) is a technique that involves the capture of all protein-coding regions of the human genome and subsequent massive parallel sequencing [1]. WES has been instrumental in increasing the diagnostic yield of rare diseases in recent years. It is progressively becoming a standard test in medical genetics units, as a natural successor to targeted NGS panels, though with increased diagnostic yield. WES is also widely regarded as a more economically efficient alternative to short-read Whole Genome Sequencing (WGS) [2,3,4]. WES interrogates regions that are readily interpretable in medical genetics, reducing costs by 3–5 times through minimizing NGS data generation, analysis, and storage, while maintaining strong diagnostic utility [5]. However, the capture-based nature of WES results in a more uneven coverage due to factors such as suboptimal enrichment of regions with extreme GC content, repetitive sequences, or high sequence identity due to homology [6,7]. Coverage uniformity is critical to not only minimize the sequencing throughput (and associated costs) required to achieve reliable variant detection, but also to ensure robust CNV imputation from WES data [7,8,9].

In recent years, commercial providers have significantly improved their probe designs to capture coding regions more comprehensively and efficiently. Comparative studies have shown how these improvements translate into greater sensitivity for SNV and indel detection, reduced PCR duplication rates, and enhanced coverage uniformity. Large-scale evaluations of commercial WES kits have highlighted that all major kits can achieve high target coverage, but performance varies across metrics such as coverage evenness, on-target rate, and precision in variant calling [3,10,11,12].

Despite these technical advances, relatively few studies have examined the impact of DNA sources and quality on WES performance in the context of rare diseases testing. Depending on the tissue origin, collection conditions, and extraction method, DNA may be overfragmented or contaminated, potentially leading to lower sensitivity. Although peripheral blood is considered the standard source of genomic DNA for clinical WES, poor sample handling can compromise DNA integrity, leading to reduced library complexity and less coverage [13,14]. Similarly, prenatal samples, including amniotic fluid or chorionic villi, have been successfully used in numerous studies [15,16,17]. Nevertheless, they present specific technical challenges such as limited DNA quantity, variable DNA quality, maternal contamination, and in some cases requirement for cell culture, which can increase turnaround time. Finally, dry bloodspots represent an alternative when conventional blood samples are unavailable; however, the limited amount of input DNA and the effects of long-term storage or suboptimal environmental conditions may result in increased DNA fragmentation and lower sequencing performance [18,19].

This study aims to compare the performance of different commercial WES capture kits using real world clinical sample tissues. We focused on specific coverage and additional quality metrics, as well as its robustness to variable input DNA quality.

## 2. Results

In this study, we aimed to compare three different, extensively validated whole exome sequencing kits currently used in molecular diagnostics settings in western Europe, namely Agilent SureSelect Human All Exon V8, Illumina DNA Prep with Exome 2.5 Enrichment, and Twist Exome 2.0 Plus Comprehensive Spike-in. We chose to test our aim using a two-step strategy, in which we first evaluated the technical performance of WES kits by sampling eight samples including four peripheral blood (PB) samples and four additional samples from four independent non-peripheral blood sources (amniotic fluid, cultured cells, and prenatal tissue) (Table 1), followed by a validation phase with a larger number of samples.

First, we compared vendor BED files containing the target regions covered by each manufacturer. While Agilent’s capture probe technology uses 80-mer ssRNA probes, Illumina DNA Prep 2.5 and Twist Exome 2.0 Plus Comprehensive Spike-in use the same 120-mer dsDNA probes. Therefore, these two kits share the same target region BED file, and the only differences between them lies in the reagents and protocol used by each kit. Overall, Illumina/Twist kits target a slightly larger portion of the genome than Agilent (37.8 Mb vs. 35.1 Mb). Also, Illumina/Twist covered 965 regions (including whole gene exonic regions) that were not present in Agilent’s BED file. In contrast, only six exonic regions were exclusively present in Agilent’s file.

**Figure 1:**
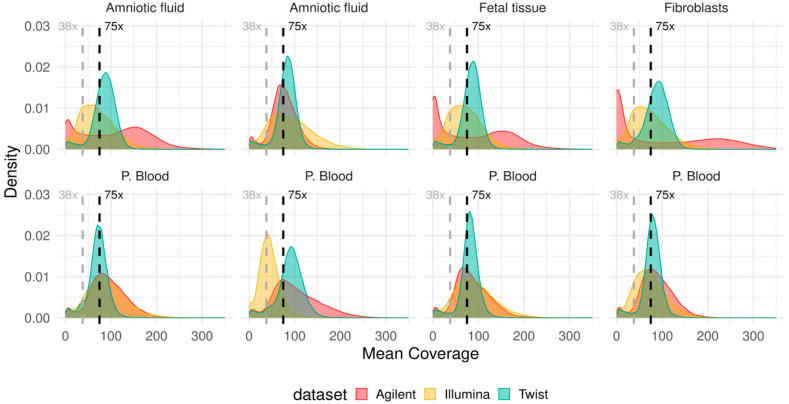
Twist Exome 2.0 WES commercial solution provides the highest coverage of exonic regions at diagnostic quality threshold (38x) while limiting oversequencing of well-covered regions (75x). X axis represents mean coverage of exonic regions, Y Axis represents density as a quantitative measure for coverage. 38x threshold is used as quality threshold as proven to be the minimal coverage to detect heterozygous variants with 99.9%. probability when VAF filter is set at 25% [20]. Sample tissue origin is indicated above each density plot.

Results from this initial comparison showed that the Illumina and Agilent library preparation kits often produced sparse coverage distributions, indicating substantial variability with both poorly and oversequenced targeted exonic regions. In contrast, the Twist Exome 2.0 kit consistently achieved a more uniform coverage across most targeted exonic regions in all samples while maintaining a high mean coverage depth. These differences were most pronounced in the non-PB samples, where the Illumina and Agilent kits struggled to reach sufficient diagnostic coverage threshold (20x) across all regions of interest (Figure 1).

To further validate quality metrics detected in the comparison phase, we sequenced 24 additional samples with Twist Exome 2.0 kit, which had previously been sequenced with Agilent SureSelect V8 kit for clinical purposes. This validation set comprised 12 PB and 12 non-PB specimens (Table 1). To standardize the comparison within a clinical framework, we generated a unified BED file containing the coding regions of 4100 clinically relevant genes curated in PanelApp database. Metrics were computed across all available datasets (Agilent, *n* = 32; Twist, *n* = 32; Illumina *n* = 8).

**Figure 2:**
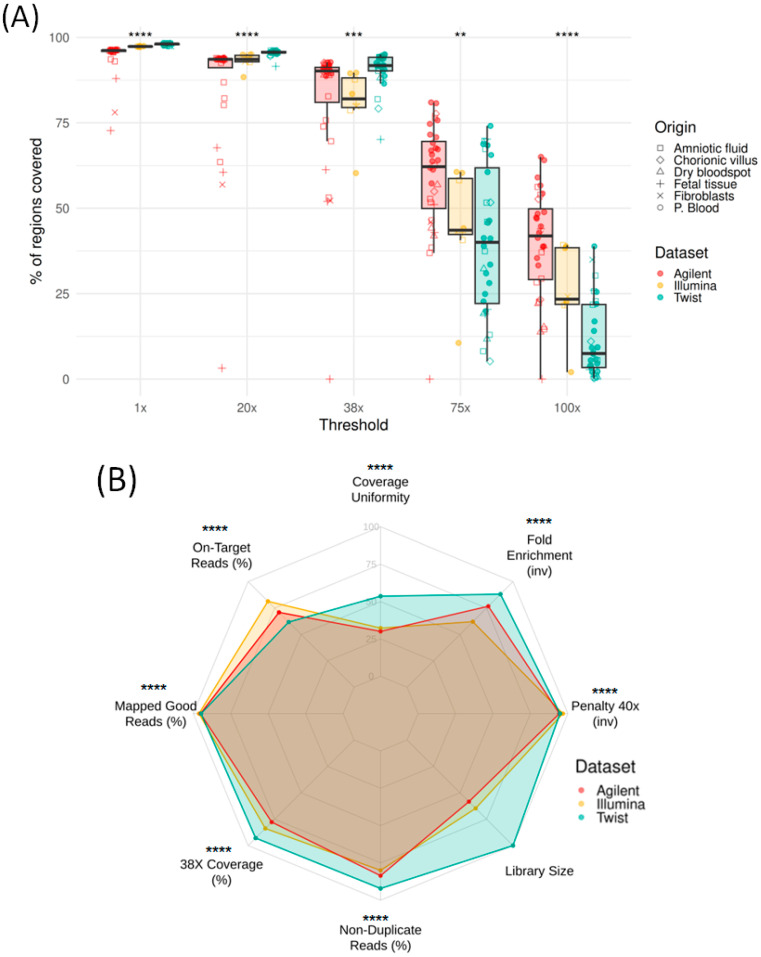
Coverage and quality features for the three WES library protocols evaluated in this study. (**A**) Boxplot summarizing % of clinically relevant covered regions at specific coverage thresholds for all datasets included in this study (*n* = 72; Twist: *n* = 32, Agilent: *n* = 32, Illumina: *n* = 8). (**B**) Radar plot summarizing quality control features among the three different libraries, for all datasets in this study. Metrics used are detailed in Materials and Methods section. Statistical significance was defined as *p*-value < 0.05 and denoted as ** (*p* ≤ 0.01), *** (*p* ≤ 0.001), and **** (*p* ≤ 0.0001).

When comparing coverage metrics using the set of target regions, the Twist Exome 2.0 kit consistently delivered a higher percentage of covered exonic regions at critical thresholds (1x/20x/38x). This superior coverage was evident in PB samples (mean coverage at 38x; Agilent: 91.05%; Illumina: 80.74%; Twist: 92.42%), and was specially pronounced for non-PB samples, where the other kits clearly underperformed (mean coverage at 38x; Agilent: 72.15%; Illumina: 81.66%; Twist: 88.98%). Additionally, the Twist Exome 2.0 solution exhibited less oversequencing at high depth thresholds (75x and 100x) compared to the other kits (Figure 2A, Supplementary Table S1).

Assessment of NGS quality indicators confirmed the robust performance of the Twist Exome 2.0 kit, which provided superior results for fold enrichment, library size, coverage uniformity, percentage of duplicates, and coverage of clinically relevant regions at 38x. Performance metrics for correct read mapping and Penalty 40x remained similar across all kits (Figure 2B, Supplementary Table S1). Twist Exome 2.0 solution demonstrated better performance than competitors for both PB and alternative tissue types (Supplementary Figures S1–S6). On the other hand, Twist kit was shown to underperform at on-target-reads percentage compared to other kits, possibly linked to a higher capture of near-target regions [21].

Finally, we evaluated the performance of each kit in difficult-to-sequence (DTS) regions, which are challenging due to features such as high GC content, low complexity, or sequence homology. We focused on a set of 795 clinically relevant regions (representing 175 unique genes) out of a total of 410,966 regions belonging to 1235 genes described by Hijikata and colleagues recently [22]. The successful capture of these 795 regions serves as a critical indicator of each platform’s robustness in acquiring challenging, yet diagnostically essential, genomic content. We calculated the number of these regions where at least 95% of the length was covered at the 38x threshold [20] (Table 2). The results demonstrated a clear advantage for the Twist Exome 2.0 kit, which successfully covered an average of 23.3% of the 795 DTS regions. In contrast, the competing libraries achieved significantly lower performance, covering an average of 14–16% of these regions.

## 3. Discussion

In this study, we compared three WES solutions frequently used in labs and provide evidence suggesting that Twist WES Exome 2.0 significantly outperformed its competitors. This superior performance is particularly evident in samples with compromised sequencing quality associated with prenatal or alternative tissues. Our findings align with a previous study that reported an increased Twist performance, especially for low-variant allele frequency analysis [23]. Although comprehensive studies on WES performance on DNA extracted from peripheral blood have been recently published [10,12], the literature about WES applied on other non-blood matrices (specially low-input, degraded samples) is scarce. Nevertheless, previous studies have been published emphasizing that Twist WES solution is effective on DNA extracted from solid tissues such as FFPE biopsies or dried bloodspots in terms of higher uniformity, or whole exome coverage compared to other solutions [24,25,26]. To our knowledge, our manuscript is the first to benchmark the performance of different WES solutions on prenatal materials such as amniotic fluid or chorionic villi. Although WES applied to prenatal samples’ DNA has greatly expanded in recent years, in our experience the preanalytic factors or different extraction methods’ impact on DNA quality, and thus effect of using different WES solutions to tackle these difficulties, is relevant [27,28]. Although recent studies have successfully used long-read sequencing for degraded or low-input DNA samples, many samples still might not benefit from these emerging sequencing technologies, and thus optimization of short-read methods for quality-compromised tissues will still be required [21,29,30].

The enhanced uniformity and superior coverage at diagnostic thresholds (38x), particularly in difficult-to-sequence regions, is expected to increase the diagnostic yield. This improved data quality is critical for maximizing sensitivity in detecting single nucleotide variants (SNVs) and small indels, and for ensuring the fidelity of copy number variant (CNV) detection, which relies heavily on uniform coverage [10,31,32]. Importantly, these relative differences in uniformity, off-target reads, or coverage observed between different WES kits are largely driven by the enrichment chemistry, and are therefore expected to remain stable when alternative secondary analysis pipelines are applied [12,33]. From a laboratory perspective, the superior performance of the Twist Exome 2.0 kit was achieved while increasing the number of samples per run and decreasing hands-on time and per-sample reagent cost compared to former solutions used in our lab, making the data quality improvements a cost-effective choice for clinical laboratories (Supplementary Table S3).

While Twist solution was superior in most evaluated aspects, both Agilent and Illumina were superior regarding on-target reads rate. Given that Illumina and Twist kits use an identical BED file, the greater on-target capture efficiency of the Illumina kit indicates that factors other than target design, such as probe chemistry, are responsible for the observed performance differences. A possibility is that, as Twist protocol has been reported to capture longer exon-adjacent regions than other WES solutions, capturing and sequencing these regions result in a higher off-target percentage [34]; however, we cannot exclude off-targets in the rest of the genome and a careful evaluation of that phenomenon is needed. As Illumina and Twist WES metrics differ substantially while probe chemistry and design are the same, our work illustrates the importance of optimizing library generation and capture protocols, and hint to the need of future improvements for these WES kits [35,36,37].

WES is increasingly being adopted as a first-tier test for its greater benefits compared to targeted gene panels [38,39,40] as well as being used before/in parallel to additional screening methods [41]. However, given the increased diversification of the WES market (>15 commercial solutions available), there is an urgent need to agree on a common set of evaluation metrics and define some standards to objectively evaluate the different solutions. First, the heterogeneity of BED files across different kit manufacturers makes it challenging to determine whether all relevant exonic regions are adequately targeted. While RefSeq or Ensembl exonic regions are used for benchmarking, a universal consensus is needed regarding what constitutes a “whole exome”. Second, as the number of genes associated with medical conditions and rare diseases continues to grow, including non-protein-coding genes or genes with variants which are not easily detected by short-read NGS [42,43], there is no consensus either on which genes and regions should constitute the “clinical exome”. Consequently, the definition is left to commercial kit providers. Collaboration frameworks such as GenCC or PanelApp are crucial to establish what genes are unequivocally linked to human disease while specifying different levels of evidence [44,45,46]. Third, although mean coverage is increasingly understood to be a less critical metric than coverage uniformity (e.g., 95% at 20x), this distinction remains unfamiliar to many clinical and non-technical staff. Given its importance on optimizing WES costs and improving features such as CNV calling, the use and dissemination of coverage-related metrics is key [7,47]. Finally, while whole exome global coverage is a relevant metric, it is also very crucial to know which hard-to-sequence, medically relevant regions are not effectively covered by each WES solution [7,12]. The definition of these standards would help to better compare WES solutions and complement choice based on wet-lab features such as compatibility to sequencing platforms, protocol requirements, automatization, turnaround time, and pricing among others.

In this study we have encountered several limitations. First, we evaluated a limited number of DNA samples, particularly those extracted from diverse non-peripheral blood matrices such as prenatal samples. These samples were grouped into a single “non-PB” category rather than analyzed separately by tissue type. Therefore, caution should be applied when generalizing the results obtained for these alternative matrices in this study. Another limitation is that the validation phase (*n* = 24) did not include Illumina’s kit. This decision was based on the initial phase results, where this kit showed lower coverage at clinical thresholds compared with the other two solutions, and particularly compared to Twist’s kit, which used the same 120-mer dsDNA probes. Regarding library sequencing, Twist libraries were sequenced with 2 × 150 bp reads, whereas the other two kits were sequenced with 2 × 100 bp. This difference reflects our decision to strictly follow the official protocols and recommendations provided for each WES solution. Although this variation may influence results due to differences in read mappability [34], we expect that overall coverage and performance metrics would have remained comparable. In addition, we did not assess additional commercial WES solutions with larger target region sizes such as those offered by Roche, MGI, or IDT, which may include near-exonic or non-coding regions. However, a more comprehensive design does not necessarily translate into uniform, sufficient coverage across all WES targeted regions. These solutions should therefore be properly benchmarked in future studies. Finally, in this work we have not leveraged the use of reference samples such as Genome in a Bottle or alternative synthetic methods [23,48], as the primary focus was on DNA samples derived from tissues processed in a daily basis in clinical facilities.

In summary, our study suggests that Twist Bioscience Exome 2.0 provides robust performance in terms of coverage and uniformity, and is particularly well-suited for low DNA quality samples such as prenatal samples, cultured cells or dry bloodspots. Furthermore, the field would benefit from consensus-driven guidelines for benchmarking methodologies and standardized reporting metrics, which would empower clinical laboratories to objectively evaluate and select the optimal platform for their needs.

## 4. Materials and Methods

### 4.1. DNA Extraction

Genomic DNA was extracted using different procedures depending on the tissue of origin. For peripheral blood, genomic DNA (gDNA) was extracted using the MagNA Pure 96 DNA and Viral NA Large Volume Kit on the MagNA Pure 96 Instrument (Roche Diagnostics, Basel, Switzerland).

For cell pellets, gDNA was isolated using the Maxwell^®^ CSC Genomic DNA Kit on the Maxwell^®^ CSC automated nucleic acid extraction system (Promega Corporation, Madison, WI, USA). Both extraction protocols were performed at the Molecular Biology Core Laboratory, Biomedical Diagnostic Center, Hospital Clínic de Barcelona, following the respective manufacturers’ instructions.

For amniotic fluid, chorionic villus, and other solid prenatal samples, gDNA was extracted using a standard protocol based on proteinase K digestion followed by phenol/chloroform extraction. Briefly, samples were incubated with lysis buffer and proteinase K at 56 °C until complete digestion. DNA was then purified by sequential extraction with phenol and phenol/chloroform/isoamyl alcohol, followed by ethanol precipitation. The DNA pellet was washed, air-dried, and resuspended in TE buffer.

Genomic DNA extraction from dried blood spots was performed using the Prepito DNA Cyto Pure Kit (Revvity, Waltham, MA, USA). Three paper disks of 3.2 mm in diameter were punched for each sample. Lysis buffer, proteinase K, and dithiothreitol (DTT) were added to the samples, followed by incubation and agitation at 56 °C for 1 h. After incubation, the samples were centrifuged, and the resulting supernatant was transferred to a 96-well plate. Automated DNA extraction was then carried out using the chemagic Prepito-D instrument (Revvity) according to the manufacturer’s protocol.

All DNA samples were checked for concentration and quality using Qubit™ Fluorometer and Nanodrop instruments (Thermo Fisher Scientific, Waltham, MA, USA) according to the manufacturer’s instructions. All samples were normalized and diluted so that the input DNA quantity was 100 ng of dsDNA per sample.

### 4.2. Library Preparation

Three commercial library preparation kits were evaluated departing from 100 ng of gDNA and following the manufacture’s recommendations.

For Agilent SureSelect V8 kit (Agilent Technologies, Inc., Santa Clara, CA, USA), DNAs were initially fragmented by sonication using Covaris ME-220 to achieve a mean peak size of ~200 bp. Then, whole exome sequencing libraries were prepared using Magnis NGS Prep System, in batches of 8 samples. Magnis platform protocol automatizes the steps of DNA Repair, adaptor ligation, indexing, DNA capture, and PCR enrichment. Quality control was performed using the 4200 TapeStation platform (Agilent Technologies, Santa Clara, CA, USA). A total of 11 PCR cycles were performed for indexing, while 10 were done for PCR enrichment after DNA capture. DNA concentration was measured using Qubit^®^ instrument (Life Technologies, Carlsbad, CA, USA) with the dsDNA HS Assay Kit.

For Illumina DNA Prep with Exome 2.5 Enrichment (Illumina, Inc., San Diego, CA, USA), DNAs were tagmented to normalize DNA libraries to an average peak size of ~350 bp. Before hybridization, libraries were combined into 8-plex pools (250 ng of each library). A total of 9 PCR cycles were performed for indexing, while 12 were done for PCR enrichment after DNA capture.

For Twist Exome 2.0 Plus Comprehensive Spike-in (Twist Bioscience, San Francisco, CA, USA), DNA was initially fragmented for 10 min at 30 °C to obtain an average peak size of ~450 bp. Before hybridization, libraries were combined into 8-plex pools (250 ng of each library). Seven PCR cycles were performed for indexing, while 8 were done for PCR enrichment after DNA capture.

### 4.3. Sequencing

All libraries were sequenced on an Illumina NextSeq2000 sequencer using P3 flow cells. Libraries were 2 × 150 paired-end for Twist Bioscience Exome 2.0 exome libraries, and 2 × 100 paired-end for both Illumina DNA Prep with Exome 2.5 Enrichment and Agilent SureSelect V8.

### 4.4. Bioinformatics Analysis

Clinical region selection: Original target regions BED files for all three kits were obtained directly from the vendors in GRCh38 reference genome. To assess coverage of clinically relevant regions, we selected diagnostic-grade ‘green’ genes from the 451 gene panels in PanelApp UK (consulted on 22 October 2024) [45] and generated a BED file including all the coding exons for each of the 4100 genes using Ensembl GTF annotation (v111). We computed the overlap between target regions and clinically relevant genes using BEDtools intersect v2.31.1 [49]. Additionally, a catalog of regions difficult to sequence and present in genes linked to rare human diseases was obtained from Hijikata et al., 2024 [22].

NGS data processing: We developed a comprehensive in-house bioinformatics pipeline based on Nextflow [50]. BAM files were obtained after aligning reads to GRCh38 reference genome using BWA-MEM. Duplicates were marked, base quality scores were recalibrated, and the resulting BAM files served as the input for all downstream analyses. Coverage metrics, including % of covered bases at different thresholds and coverage uniformity, were obtained using Mosdepth [51] v0.3.8 and applied to the Clinically Relevant regions BED file previously defined.

### 4.5. Metrics Calculation

Quality metrics, including duplication rates, estimated library size, percentage of on-target reads, coverage uniformity and fold enrichment, were obtained using Picard tools v3.4.0 (CalculateHsMetrics) and Samtools v1.21 (flagstats) [52] (Supplementary Table S1). All generated metrics were evaluated based on the target region BED files defined by each commercial solution.

### 4.6. Statistical Analyses and Figures

Differences in performance and coverage metrics among libraries from the three different WES kits were evaluated using the Kruskal–Wallis rank-sum test. Pairwise comparisons to observe differences between two groups were subsequently performed using Dunn’s post hoc test with Benjamini–Hochberg correction and can be found in Supplementary Table S2. Statistical significance was defined as *p*-value < 0.05 and denoted as ns (*p* > 0.05), * (*p* ≤ 0.05), ** (*p* ≤ 0.01), *** (*p* ≤ 0.001), and **** (*p* ≤ 0.0001). All statistical analyses and figures were generated using R 4.5.2 through the ggplot2 library [53].

## Figures and Tables

**Table 1:** Composition of sample sets used for (1) initial comparison of three WES commercial solutions, and (2) validation of the results, using a larger sample size for both peripheral blood and additional tissues.

	Tissue	N	WES Library Preparation Methods
WES kit comparison phase	Peripheral blood	4	- Agilent SureSelect v8 WES (Magnis platform) - Illumina DNA Prep with Exome 2.5 (Hamilton platform) - Twist Bioscience Exome 2.0 + Comprehensive exome spike-in (Hamilton platform)
	Amniotic Fluid	2	
	Fetal Tissue	1	
	Fibroblasts	1	
WES kit validation phase	Peripheral blood	12	- Agilent SureSelect v8 WES (Magnis platform) - Twist Bioscience Exome 2.0 + Comprehensive exome spike-in (Hamilton platform)
	Amniotic Fluid	5	
	Fetal tissue	2	
	Chorionic villi	2	
	Dry bloodspots	3	

**Table 2:** Assessment of clinically relevant regions resistant to short-read sequencing (n = 795) (Hijikata et al., 2025) [22].

Dataset	N Samples Assessed	Challenging Regions >95% Covered at 38x (Median [IQR])	% Challenging Regions >95% at 38x (Median [IQR])
Agilent	32	112.5 [98–127]	14.2 [12.3–16]
Illumina	8	124.5 [99.5–149.5]	15.7 [12.5–18.8]
Twist	32	185.5 [165.5–205.5]	23.3 [20.8–25.8]

## Data Availability

The data that support this study’s findings are available on reasonable request to the corresponding authors. The data are not publicly available due to privacy and ethical reasons.

## Supplementary Materials

The following supporting information can be downloaded at: https://www.mdpi.com/article/10.3390/ijms27146261/s1.
