# Supplementary material for: Comparison of Whole Exome Sequencing Commercial Kits Performance Across Diverse Tissue Sources
Source: Int J Mol Sci. 2026 Jul 14;27(14):6261. doi: 10.3390/ijms27146261 (PMC13409959; doi:10.3390/ijms27146261)

SUPPLEMENTARY MATERIAL

Supplementary Table S1: Summary and description of the metrics used to compare the different libraries used.

Metric	Origin	Definition
1x, 20x, 38x, 75x, 100x Coverage	Mosdepth	% of bases covered at a certain threshold
Mean Coverage	Mosdepth	Average number of sequencing reads that align to each base within the target region of a sample
Coverage Uniformity	Mosdepth	Proportion of target bases whose coverage depth fell within a ±20% range around the mean coverage of the sample
Total Reads	Samtools	Total number of sequencing reads obtained
Mapped Good Reads (%)	Samtools	Proportion of sequencing reads that are both successfully aligned to the reference genome and pass quality filters
On-Target Reads (%)	Samtools	% of reads that correctly map to a set of regions of interest
Duplicate Reads & Non-Duplicate Reads (%)	Samtools	% duplicate reads and its Inverse, refer to reads that align to the exact same genomic location as other reads, meaning they originate from the same original DNA fragment
Read Pairs Examined	Picard tools	Number of mapped read pairs evaluated
Library size	Picard tools	Estimation of the number of fragments or reads in a prepared
Fold enrichment	Picard tools	Ratio of the average coverage in the target regions to the average coverage in the non-target (background) regions
Penalty 40x	Picard tools	Quantifies how much more sequencing is required due to insufficient or uneven coverage across target regions and excess reads in off-target regions

Supplementary Table S2: Excel file containing metrics for all 32 Whole Exome Sequencing datasets generated in this study using 3 different vendors (joint file)

Supplementary Figure S1: WES coverage by sample at different thresholds, for the 3 WES kits evaluated. Y-axis: % of covered regions. X-axis: 1/20/38/75/100x coverage thresholds

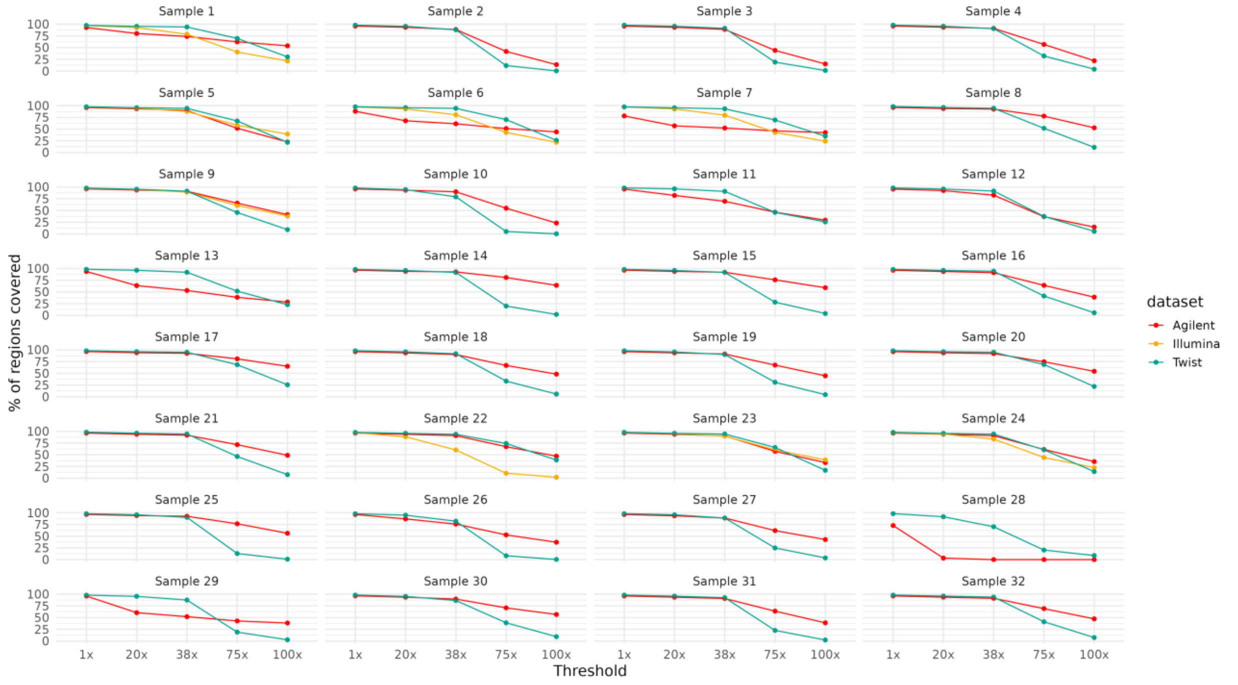

Supplementary Figure S2: WES estimated library sizes by sample, for the 3 WES kits evaluated.

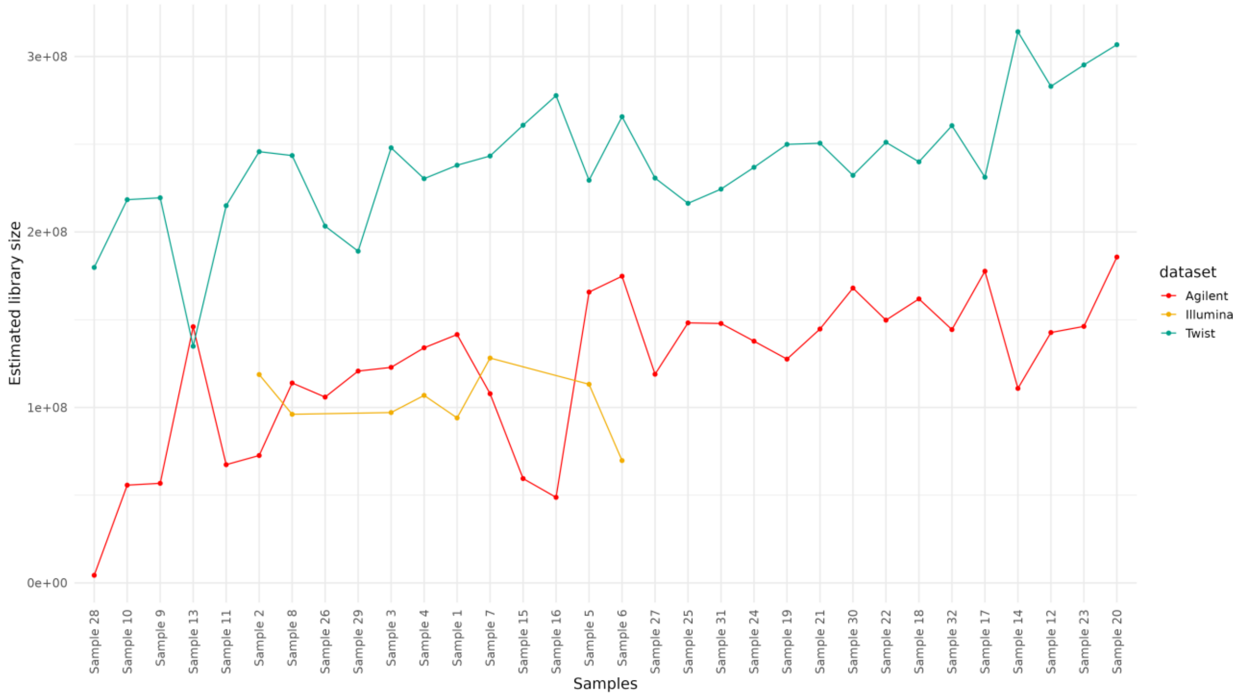

Supplementary Figure S3: WES duplicates rate (fraction of total reads) by sample, for the 3 WES kits evaluated.

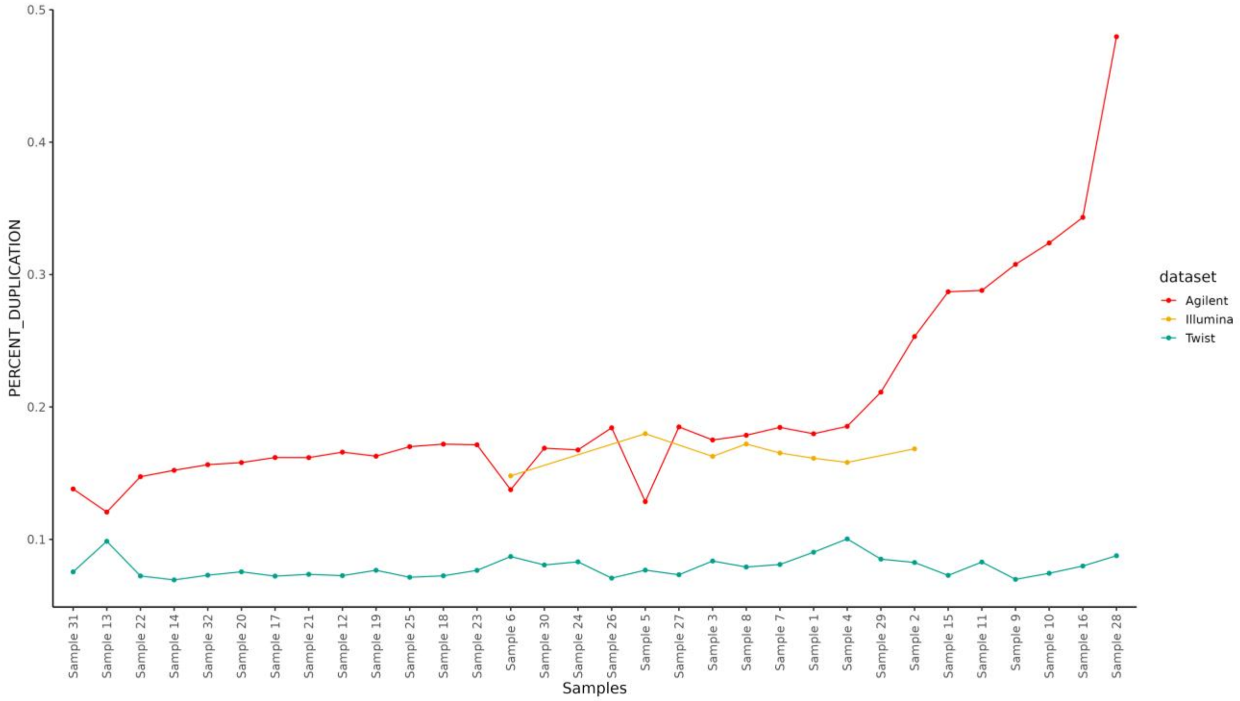

Supplementary Figure S4: WES read pairs by sample at different thresholds, for the 3 WES kits evaluated.

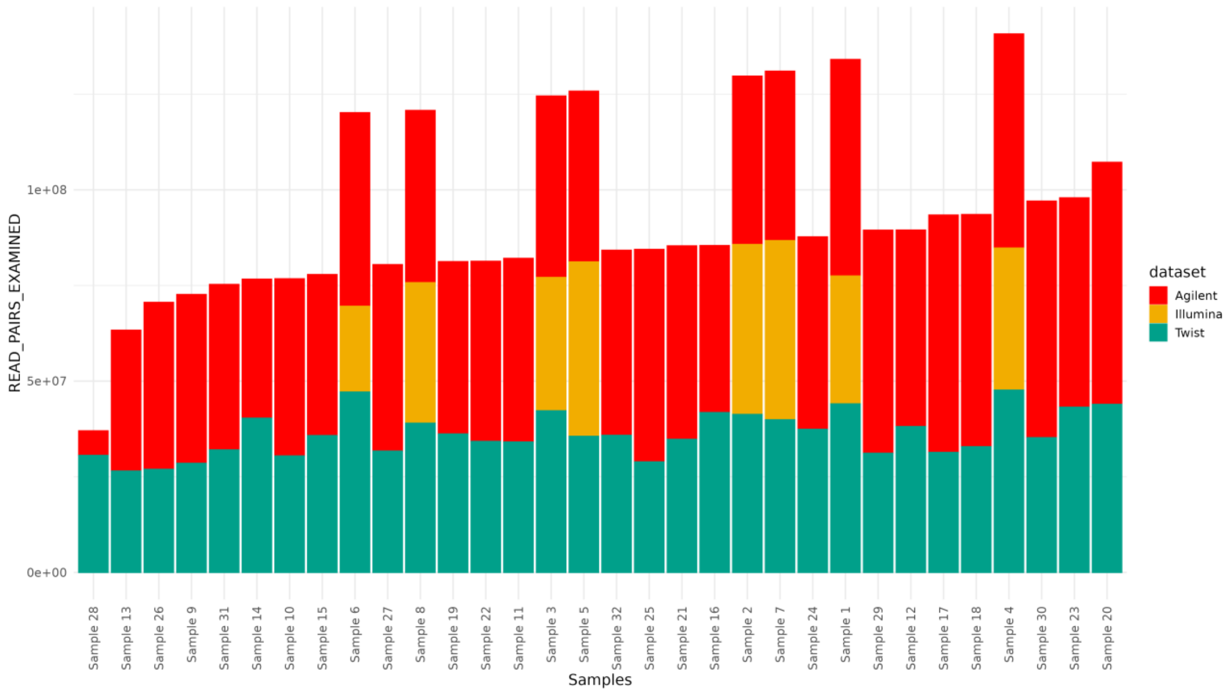

Supplementary Figure S5: Radar plots displaying WES features for peripheral blood samples (n=16)

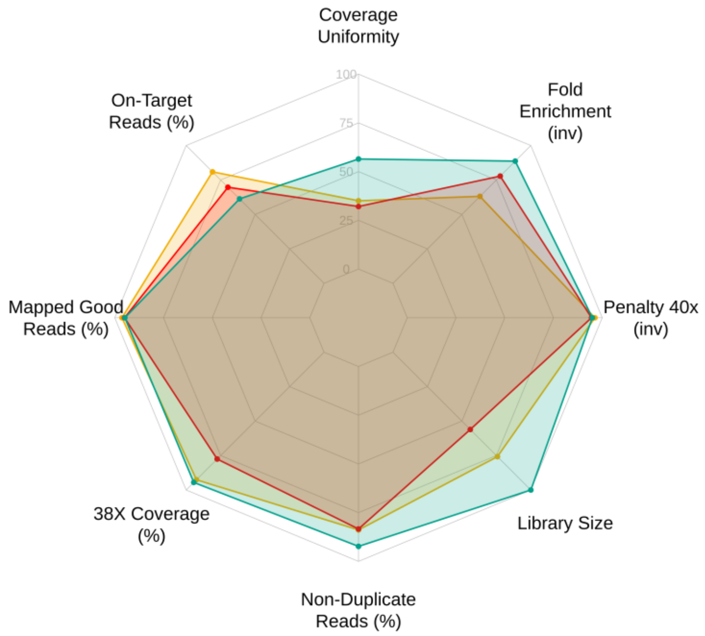

Supplementary Figure S6: Radar plots displaying WES features for non-peripheral blood samples (n=16)

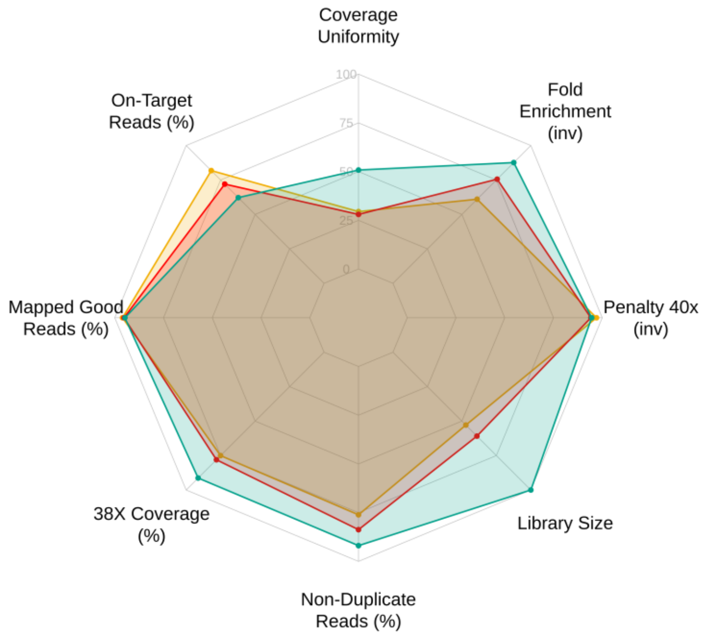

Supplement: Supplementary file 1 [file ijms-27-06261-s001.zip › SUPPLEMENTARY MATERIAL.pdf]
